# NarL, a Novel Repressor for CYP108j1 Expression during PAHs Degradation in *Rhodococcus* sp. P14

**DOI:** 10.3390/ijms21030983

**Published:** 2020-02-01

**Authors:** Jie Kan, Tao Peng, Tongwang Huang, Guangming Xiong, Zhong Hu

**Affiliations:** 1 Department of Biology, Shantou University, Shantou 515063, China; 13jkan@stu.edu.cn (J.K.); tpeng@stu.edu.cn (T.P.); twhuang@stu.edu.cn (T.H.); 2 Institute of Toxicology and Pharmacology for Natural Scientists, University Medical School Schleswig-Holstein, 24103 Kiel, Germany; xiong-long@hotmail.com

**Keywords:** repressor, LuxR, PAHs, *Rhodococcus*, palindromic sequences

## Abstract

*Rhodococcus* sp. P14 was isolated from crude-oil-contaminated sediments, and a wide range of polycyclic aromatic hydrocarbons (PAHs) could be used as the sole source of carbon and energy. A key CYP450 gene, designated as *cyp108j1* and involved in the degradation of PAHs, was identified and was able to hydroxylate various PAHs. However, the regulatory mechanism of the expression of *cyp108j1* remains unknown. In this study, we found that the expression of *cyp108j1* is negatively regulated by a LuxR (helix-turn-helix transcription factors in acyl-homoserine lactones-mediated quorum sensing) family regulator, NarL (nitrate-dependent two-component regulatory factor), which is located upstream of *cyp108j1*. Further analysis revealed that NarL can directly bind to the promoter region of *cyp108j1*. Mutational experiments demonstrated that the binding site between NarL and the *cyp108j1* promoter was the palindromic sequence GAAAGTTG-CAACTTTC. Together, the finding reveal that NarL is a novel repressor for the expression of *cyp108j1* during PAHs degradation.

## 1. Introduction

As natural environmental products, polycyclic aromatic hydrocarbons (PAHs) have been present on the earth for many years [1]. The rapid development of industry technology and the increase in anthropogenic activities have resulted in the production of a large number of PAHs [2]. The physical and chemical properties of PAHs mean they do not degrade easily in the environment, existing ubiquitously in the air, soil, and water [3]. These PAHs can bioaccumulate through food chains, which poses a potential hazard to human health [4,5,6,7]. Bioremediation is considered a useful and available cleanup strategy and much scientific work has focused on analysis of the bacterial catabolism of PAHs [8,9,10]. In the last few decades, research on microbial degradation of PAHs has advanced significantly and a number of PAH-degrading isolates have been reported [1,11,12,13], with some of the isolates belonging to the *Rhodococcus* genus.

*Rhodococcus* can degrade many organic compounds. They are ideal candidates for enhancing the bioremediation of contaminated sites and have been proven useful for a wide range of biotransformations, such as PAHs, steroid modifications, enantioselective synthesis, and the production of amides from nitriles [14]. Water et al. used pyrene as a sole source of carbon and energy and isolated the *Rhodococcus* sp. UW1 from contaminated soil, which mineralized 72% of the pyrene within two weeks. At pH 7.0 and 30 °C, it showed a maximum degradation rate of 0.08 mg pyrene/mL per day. *Rhodococcus* sp. UW1 has a broad substrate spectrum; phenanthrene, anthracene, fluoranthene, and chrysene can also be used as sole sources of carbon and energy [15]. A common feature of the aerobic *Rhodococcus* genus is the presence of many types of monooxygenases and dioxygenases. Cytochrome p450 (CYP450) plays an important role in the process. Sylvie et al. characterized spontaneous mutants of *Rhodococcus ruber* unable to use ethyl tert-butyl ether (ETBE) as the sole source of carbon and energy and found that it was unable to degrade ETBE without a CYP450 gene cluster, whereas the complementation of the mutant using *ethRABCD* was able to degrade ETBE again, demonstrating the involvement of the Eth CYP450 system in the degradation of ETBE [16].

Regulatory proteins and regulated promoters are key elements that control the transcription of catabolic substrates, such as PAHs [17]. The regulator DfdR is one of the LuxR family proteins, which is encoded by a gene in the *dfd* gene cluster in the dibenzofuran using *Rhodococcus* sp. strain YK2 and *Terrabacter* sp. strain YK3. The *dfdR* gene product affects the promoter activity of the *dfdA* gene, which is involved in the initial hydroxylation of dibenzofuran [18].

*Rhodococcus* sp. P14 was originally isolated from crude-oil-contaminated sediments and can use a wide range of PAHs and steroids as the sole source of carbon and energy [19,20,21]. Its entire genome has been sequenced [22]. Some hydroxyl products were detected during *Rhodococcus* sp. P14 metabolism of PAHs, suggesting that oxygenase plays an important role in the degradation process [23,24]. The gene *cyp108j1* encoding a CYP450 was identified. Further investigation of the recombinant protein CYP108J1 proved that it is capable of the hydroxylation of a series of PAHs compounds [24].

In this study, we analyzed the promoter structure of *cyp108j1* and proved that its upstream regulatory NarL has an inhibitory effect on the *cyp108j1* expression. Using site-directed mutation, we identified the binding sites of NarL with the promoter *cyp108j1*. These findings can help us to understand more about the regulation mechanism during PAHs degradation in microorganisms.

## 2. Results

### 2.1. Indentified Promoter of CYP108J1

**Figure 1:**
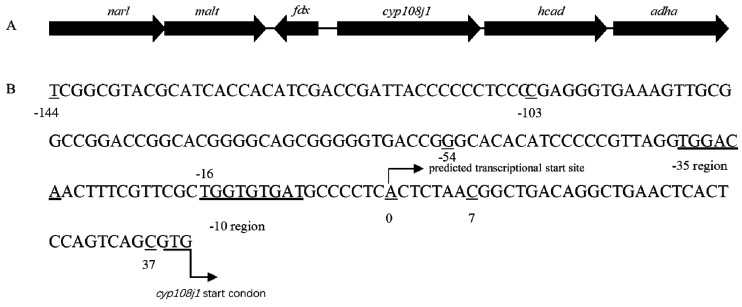
(**A**) The genomic organization of gene cluster containing *cyp108j1.* (**B**) The promoter P3 of *cyp108j1*.

In previous study, we found that CYP108J1 is capable of high molecular weight PAHs oxidization and plays an important role in PAHs degradation in *Rhodococcus* sp. P14. In this gene cluster, two same-orientation LuxR family transcriptional regulatory genes (*narl* and *malt*) were identified upstream of *cyp108j1* (Figure 1A). Using Softberry software, one promoter in the 182 bp intergenic region in the upstream of *cyp180j1* was predicted and named P3. The −35 bp region, −10 bp region, and transcriptional start site (TSS) were also predicted (the predicted transcription site A was set to 0) (Figure 1B).

**Figure 2:**
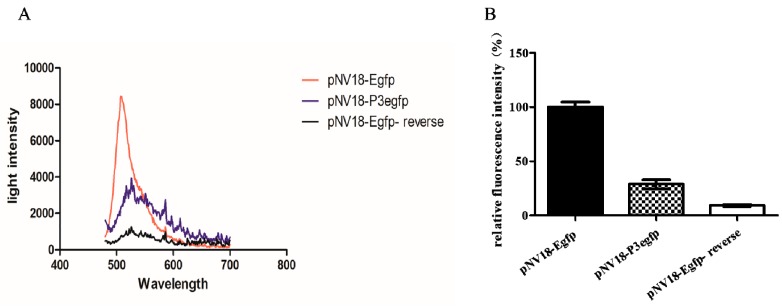
Detection of the function of P3 promoter. (**A**) The fluorescence detection of these *Escherichia coli* with pNV18-P3Egfp by a fluorescence spectrometer from 480 to 700 nm. (**B**) The fluorescence detection of the *E. coli* strains with pNV18-P3Egfp, pNV18-Egfp, and pNV18-Egfp-reverse at 510 nm. The fluorescence intensity of pNV18-Egfp was set to 100%. The error bars indicate the standard deviation.

To detect whether promoter P3 was functional, the DNA sequence including the P3 promoter (182 bp from −144 to 37 bp as shown in Figure 1B) was fused with *egfp* in the plasmid pNV18 reverse, resulting in plasmid pNV18-P3Egfp (*egfp* can only be transcribed from the P3 promoter), which was transferred into *Eschericia coli.* The *E. coli* strain with pNV18-P3Egfp showed the fluorescence intensity, indicating that the P3 promoter was functional (Figure 2A). The *E. coli* strain with pNV18-Egfp-reverse was used as the negative control (*egfp* was inserted into the opposite direction of the *lac* promoter; therefore, it could not be transcribed) and pNV18-Egfp was used as the positive control (*egfp* can be transcribed from the *lac* promoter in plasmid) (Figure 2B).

**Figure 3:**
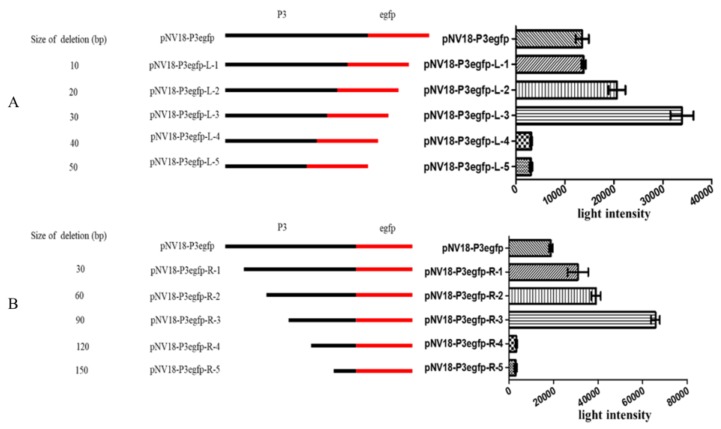
The activity analysis of various lengths of the P3 promoter. (**A**) The activity detection of the P3 promoter with deletion of 10 to 50 bp from the 3′ terminus. (**B**) The activity detection of the P3 promoter with deletion of 30 to 150 bp from the 5′ terminus. The deleted fragments of the P3 promoter were inserted into the plasmid pNV18 reverse and transferred into *E. coli* to detect the fluorescence. All data are presented as means ± standard deviation (error bars).

For localization of promoter P3, various deleted fragments of the 182 bp sequence were linked with *egfp* directly and incorporated into the plasmid pNV18 reverse (*egfp* can only be transcribed from the P3 promoter), resulting in 5′ set and 3′ set plasmids (Table S1, Figure 3). These recombinant plasmids were transformed into *E. coli* DH5α to detect the fluorescence intensity. The *E. coli* with pNV18-P3Egfp was used as a control. As the deletion of 10 to 30 bp from the 3′ terminus and deletion of 30 to 90 bp from the 5′ terminus produced 1.02- to 2.5-fold and 1.64- to 3.49-fold higher fluorescence intensity than the control, respectively. With deletion of 120 bp from the 5′ terminus and 40 bp from the 3′ terminus, the fluorescence intensity decreased dramatically (Figure 3). These results agreed with the Softberry analysis results, which proved that the promoter was located between −54 and +7 bp of *cyp108j1* TSS (Figure 1B).

### 2.2. NarL as a Repressor for Expression of *cyp108j1*

**Figure 4:**
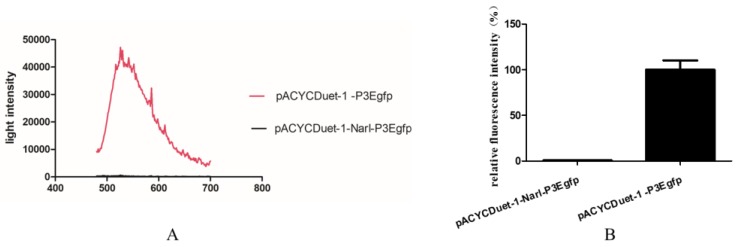
Analysis of the effect of NarL on P3 promoter. (**A**) The fluorescence detection of two *E. coli* strains with pACYCDuet-1-P3Egfp and pACYCDuet-1-NarL-P3Egfp using a fluorescence spectrometer from 480 to 700 nm. (**B**) The fluorescence detection of two *E. coli* strains with pACYCDuet-1-P3Egfp and pACYCDuet-1-NarL-P3Egfp at 510 nm. The fluorescence intensity of pACYCDuet-1-P3Egfp was set to 100%. All data are presented as means ± standard deviation (error bars).

**Figure 5:**
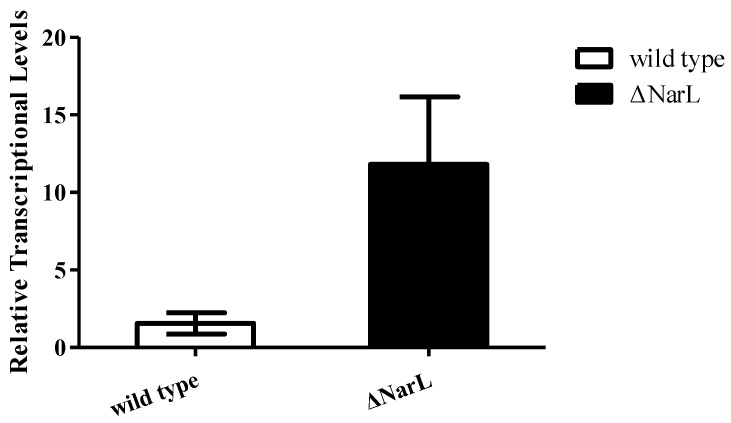
Detection of the mRNA levels of *cyp108J1* in wild type and ΔNarL cultured with benz[a]anthracene compared to that in wild type and ΔNarL cultured without benz[a]anthracene. All data are presented as means ± standard deviation (error bars).

To investigate whether NarL can modulate *cyp108j1* expression in *Rhodococcus* sp. P14, one plasmid containing the transcriptional fusion of the promoter P3 to the *egfp* was constructed (pACYCDuet-1-P3Egfp). The NarL protein was also transcribed from a T7 promoter in the same plasmid and the plasmid was named pACYCDuet-1-NarL-P3Egfp. The results showed that if the NarL was expressed in the cell, the fluorescence intensity decreased (Figure 4A,B), which indicates that NarL has a negative effect on the promoter activity of P3. The same experiment was performed for MalT, but no effect was observed. To further prove the influence of NarL on the expression of *cyp108j1*, a NarL deletion strain of *Rhodococcus* sp. P14 was constructed and named ΔNarL, which was confirmed by polymerase chain reaction (PCR) as shown in Figure S1. The expression levels of *cyp108j1* in the wild type and ΔNarL cultured with one typical PAHs benz[a]anthracene as the only carbon source were compared to those in these strains cultured without benz[a]anthracene. The expression level of *cyp108j1* increased by a 2.4 fold change compared with the wild type when it was cultured with benz[a]anthracene; this increase of *cyp108j1* expression was much stronger in ΔNarL (Figure 5), which confirmed that NarL, as a repressor, has a negative effect on the expression of *cyp108j1*.

### 2.3. NarL Directly Binds to the Promoter of *cyp108j1*

**Figure 6:**
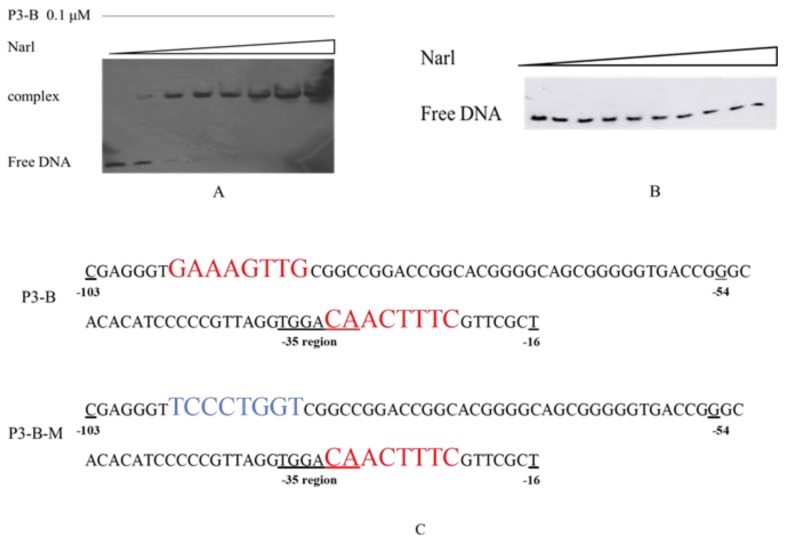
DNA-binding assays for DNA fragment and NarL. (**A**) The shift assay with NarL and P3-B. The concentration was increased from 0.05 to 2 μM. (**B**) The shift assay with NarL and P3-B-M. (**C**) The sequence of P3-B and its mutant P3-B-M. The red color sequences are the palindrome sequences. The blue sequence is the mutation sequence.

Since NarL is a regulator, the direct binding between NarL and P3 promoter was expected, and electrophoretic mobility shift assay (EMSA) was performed for confirmation. Several DNA sequences in the intergenic region were tested for their binding ability to NarL, and only one sequence, named P3-B (as shown in Figure 1B from −103 to −16 pb), could produce the binding complex with NarL (data not shown). The results showed that there was no shift band when the concentration of NarL was low (0.05 and 0.1 μM); however, one shift band was observed as the concentration of NarL increased from 0.2 to 2 μM (Figure 6A). The competition experiments confirmed that the binding between NarL and P3-B was a specific interaction (Figure S2).

A special palindrome sequence GAAAGTTG-CAACTTTC was identified from −96 to −88 and from −30 to −22 in the P3-B; one of these two sequences CAACTTTC covered both −10 and −35 regions (Figure 6C). As the only palindrome sequence in the P3-B, we propose that this structure might be related to the binding of NarL protein. To further test our hypothesis, a mutated DNA fragment of P3-B, named P3-B-M (Figure 6C), was designed for EMSA analysis. If the palindrome sequence GAAAGTTG was mutated to TCCCTGGT (Figure 6C), no shift band was observed (Figure 6B), which proved that the palindrome sequence was necessary for the binding between NarL and P3-B. Taking these results together, NarL, as a repressor, can specifically bind to the palindromic sequences (GAAAGTTG-CAACTTTC) upstream of *cyp108j1*, resulting in lower expression of *cyp108j1*.

## 3. Discussion

According to research that can be traced back to the 1940s, a central role is played by the CYP450 monooxygenase system [25] in the degradation of a wide variety of foreign compounds such as environmental pollutants and drugs [26]. CYP450 receives the necessary electrons for oxygen cleavage and substrate hydroxylation from different redox partners [27]. Most electron transferred reactions begin with the electrons transferred from reduced form of nicotinamide-adenine dinucleotide (NADH) or nicotinamide-adenine dinucleotide phosphate (NADPH) and end with one oxygen atom from the terminal CYP450s into the substrate [28], which demonstrates that monooxygenase systems require multiple proteins to work together [29].

Some CYP450 monooxygenase in the CYP108 family has been reported to have the oxidization of PAHs activity. CYP108D1 from *Novosphingobium aromaticivorans* DSM12444 was reported to have the activity in the oxidation of polycyclic aromatic hydrocarbons, such as phenanthrene, biphenyl, and phenylcyclohexane [30]. CYP108A1 can effectively hydroxylate the terpene for α-terpineol oxidation [31]. CYP108N7 from *Rhodococcus* NBRC 100605 is also able to catalyze the epoxidation, hydroxylation, demethylation, and dehalogenation of low molecular weight PAHs and their products [32].

In this study, we found that other genes are located in the upstream of *cyp108j1* in the cluster. The promoters of these genes were also analyzed, and we found that the promoter of NarL, named P1, has a strong transcription ability in recombinant bacteria. The promoter of *fdx* has a weak transcription ability in recombinant bacteria (data not shown). These promoters cannot bind with NarL or MalT. The SD sequences of these three promoters are not obvious, which may be related to the expression characteristics in *Rhodococcus* [33].

Although the *cyp108j1* expression was stronger in the ΔNarL than in the wild type during PAHs degradation, we identified no significant difference in the PAHs degradation ability between ΔNarL and the wild type. This may be due to the degradation of PAHs required by the synergistic action of multiple genes and a single gene up-regulation could not improve the degradation rate of *Rhodococcus* sp. P14.

Some other studies proved that PAHs and n-alkanes can act as small molecules which can modulate the DNA-binding and regulate genes expression [34,35]. The conformation of some regulatory proteins is altered by binding small molecules, which prevent them from binding to the promoters. In this study, various degradation substrates were used to attempt release the binding of NarL to the P3 promoter of *cyp108j1*, such as acetone, biphenyl, and hydroxy-biphenyl, but none of them succeeded (Figure S3). Phosphorylation was necessary for the function of LuxR family proteins [36,37]. Many studies have proven that phosphorylation has a strong relationship with the uptake and membrane transport of hydrocarbons [38]. We predicted that NarL, as the repressor, would be released from the promoter of *cyp108j1* during PAHs degradation by an unknown mechanism; however, this still needs further study.

The CYP450 system is highly conserved in the *Rhodococcus* genus [39]. A TBLASTN search of the NCBI database revealed that *cyp108j1* and its homologues are most found in the *Rhodococcus*. Among all these *Rhodococcus*, a conserved gene cluster contains eight genes around the CYP450, which encodes enoyl-CoA hydrase (reverse), the LuxR regulatory protein, 2Fe–2S ferredoxin, CYP450, ferredoxin-NAD^+^ reductase, alcohol dehydrogenase, and aldehyde dehydrogenase (Figure S4). The two genes encoding the LuxR family regulatory proteins (NarL and MalT) which are located in the upstream of *cyp108j1* are also conserved (Figure S5A). Compared with the non-coding regions before *cyp108j1* in these gene clusters, we found the palindromic sequences CAACTTTC are also conserved (Figure S5B), which implies that this regulation of NarL on *cyp108j1* gene is widespread in the *Rhodococcus* genus.

## 4. Materials and Methods

### 4.1. Chemicals and Reagents

Benz[a]anthracene was purchased from Sigma-Aldrich (St. Louis, MO, USA). Isopropyl-d-1-thiogalactopyranoside (IPTG), T4 DNA ligase, and the restriction enzymes were purchased from TaKaRa. An RNA Extraction Kit was purchased from Promega (Promega Corporation, Madison, WI, USA). Random primers, ribonuclease inhibitor, dNTP mixture, recombinant DNase I, and SYBR^®^ Premix Ex Taq™ (Tli RNaseH Plus) were purchased from TransGen Biotech (TransGen Biotech, Beijing, China).

### 4.2. Bacteria Strains, Plasmids, and Growth Conditions

The bacterial strains and plasmids used in this study are shown in Table S1. The *Rhodococcus* sp. P14 (CGMCC NO. 2343) used in this study was isolated from crude-oil-contaminated sediments and maintained in our laboratory [19]. *E. coli* DH5*α*, BL21 (DE3), and their recombinants were grown in lysogeny broth (LB) medium at 37 °C. *Rhodococcus* sp. P14 and its NarL mutant were grown in 2216E medium [19] at 25 °C.

### 4.3. Promoter Activity Analysis

Softberry was used to identify the promoter in the upstream of *cyp108j1* (http://softberry.com). The DNA fragment included the 182 bp *cyp108j1* upstream region from ATG ligated to *egfp* and inserted into pNV18. PCR was used to generate fragments with deletions of different lengths in the 182 bp fragment upstream of *cyp108j1*. Both the 5′ and 3′ deletion fragments were linked with *egfp*. Plasmids pACYCDuet-1 with two multiple cloning sites were used to construct the co-expression system for NarL and P3Egfp. At last, these recombinant plasmids were transformed into *E. coli* DH5*α*.

The generated *E. coli* DH5α recombinants were cultured in LB medium for 12 h, until an OD_600_ of 0.6 in 37 °C. Then, cells were harvested by centrifugation at 13,500 ×*g* for 10 min, and the pellet was resuspended in 20 mM Tris-HCl (pH 7.85) buffer, followed by disruption on ice with a sonicator for 15 min (3 s sonication and 3 s rest). At last, after centrifugation at 13,500 ×*g* for 30 min, supernatant was collected and determined by the Bradford method to ensure the protein concentration of each sample was the same. A fluorescence spectrometer was used to scan the green light region of the protein sample from 480 to 700 nm, in particular at 510 nm. Calculations and statistical analyses were performed using GraphPad software [40].

### 4.4. Construction of NarL Mutant

Total DNA from *Rhodococcus* sp. P14 cells was extracted with a kit (Dongsheng Biotech Corporation, Guangzhou, China). All primers used to construct plasmids are shown in Table S2. PCR was used to generate fragments with homologous sequences both upstream and downstream of NarL. A cassette was constructed by pNV18 promoter sequences and chloramphenicol resistance gene sequences. The chloramphenicol resistance gene was amplified from pACYCDuet-1 and then inserted into plasmid pNV18 and named pNV18-cassette (Table S1). Then, a new cassette sequence, including the promoter of plasmid pNV18-cassette to the end of chloramphenicol resistance gene, was amplified by PCR and sent to BGI (BGI Biotech Corporation, Shenzhen, China) for sequencing. At last, both homologous sequences and cassette sequences were fused together and inserted into pK18mobsacb as the suicide plasmid, named pK18mobsacb-Narl (Table S1). Electrotransformation was used for pK18mobsacb-Narl transformed into *Rhodococcus* sp. P14. Chloramphenicol plates was used for the first screen. We used 2216E medium plates for the second screen. All the colonies grown on 2216E medium plates were detected by PCR for selecting the NarL mutant (Figure S1).

### 4.5. Purification of NarL

The plasmid pET-32a was used to express the protein NarL in *E. coli*. The *narL* gene sequence, which was amplified from the total DNA of *Rhodococcus* sp. P14 by PCR, was inserted into pET-32a, and then the recombinant plasmid was transformed into *E. coli* BL21 (DE3). The *E. coli* BL21 (DE3) cells with pET32a-NarL were cultured in LB medium until an OD_600_ of 0.6 at 37 °C. Then, 1 mM IPTG (final concentration) was used to induce the expression of the recombinant protein. The temperature of the medium was moved to 25 °C. After 16 h incubation, cells were harvested by centrifugation at 13,500 ×*g* for 10 min, and the pellet was resuspended in 20 mM Tris-HCl (pH 7.85) buffer, followed by disruption on ice with a sonicator for 15 min (3 s sonication and 3 s rest). The cell debris was separated from the supernatant by centrifugation and the supernatant loaded onto a nickel–nitrilotriacetic acid (Ni–NTA) agarose column (Novagen company, Madison, WI, USA) to purify the recombinant protein NarL. All proteins were deconcentrated by a protein dialysis membrane (GE Healthcare Life Sciences China, Beijing, China). Soluble NarL was assessed with sodium dodecyl sulfate-polyacrylamide gel electrophoresis (SDS-PAGE). The concentration of purified protein was determined using the Bradford method.

### 4.6. EMSA

EMSA analysis was used for detecting protein–nucleic acid interactions [41]. All the oligonucleotides containing P3-B and P3-B-M were amplified by specific primers with biotin at the 5′ terminus of the top strand (BGI Biotech Corporation, Shenzhen, China). Pure DNA fragments were obtained using a gel recovery kit (Dongsheng Biotech Corporation, Guangzhou, China) for further assays. A chemiluminescent EMSA kit (Beyotime Biotech Corporation, Shanghai, China) was used to test the labeled DNA and protein binding. The mixture sample contained 1 μL labeled DNA (0.1 μM), 2 μL purified protein NarL in the concentration as needed (from 0.05 to 2 μM in this study), and 2 μL EMSA/gel-shift binding buffer (5×), and lastly, the nuclease-free water was added to a 10 μL total. After incubation at 25 °C for 20 min, the mixture samples were separated on nondenaturing 5% polyacrylamide gels in 0.5× Tris borate ethylene diamine tetraacetic acid (EDTA) buffer (pH 8.3) for 2 h at 10 V cm^−1^ at 4 °C, and then transferred to positively charged nylon membranes for 40 min at 380 mA. All operations were performed according to the manufacturer’s instructions.

### 4.7. RNA Isolation and Quantitative Real-Time PCR

*Rhodococcus* sp. P14 and the NarL mutant grown on mineral basal medium (MBM) were supplemented with 10 μg mL^−1^ Benz[a]anthracene. Total RNA was isolated by using an Eastep^®^ Super Total RNA Extraction Kit (Promega Corporation, Madison, WI, USA) according to the manufacturer’s instructions. qPCR was performed in a 96-well plate on a Roche Light-Cycler480 system (Roche, Diagnostics, Mannheim, Germany). The 2^−ΔΔCt^ algorithm was used to determine the relative fold changes in transcript levels. *recA* was used as a reference gene [42].

## 5. Conclusions

Our findings demonstrate that NarL is a novel repressor for the expression of *cyp108j1* during PAHs degradation. This regulation mode may exist widely in the *Rhodococcus* genus. Overall, these achievements could helpful construct effective bioremediation strategies in the near future.

## Supplementary Materials

Supplementary Materials are available online at https://www.mdpi.com/1422-0067/21/3/983/s1.
