# Supplementary material for: NarL, a Novel Repressor for CYP108j1 Expression during PAHs Degradation in Rhodococcus sp. P14
Source: Int J Mol Sci. 2020 Feb 1;21(3):983. doi: 10.3390/ijms21030983 (PMC7037279; doi:10.3390/ijms21030983)
Supplement: Supplementary file 1 [file ijms-21-00983-s001.pdf]

Supplementary Material

Supplementary Tables S1–S2

Supplementary Figures S1–S5

Table S1. List of strains and plasmids used in the study.

Strain or Plasmids	Relevant characteristic(s) (genotype)	Reference or source
**Strain**		
Rhodococcus sp. P14		(Song et al., 2011)
Escherichia coli DH5α	Cloning strain	TaKaRa
E. coli BL 21 DE3	Expression strain	TaKaRa
ΔNarL	*Rhodococcus* sp. P14 without functional *narl* gene	This study
**Plasmids (cloning and expression vectors)**		
pNV18	*Rhodococcus*–E. *coli* shuttle vectors Kan^r	Keep in our lab
pET-32a	Expression plasmid, Amp^r	TaKaRa
pMD™19-T	Cloning vector, Amp^r	TaKaRa
pET-32a-Egfp	Provide *egfp* gene	Keep in our lab
pK18mobsacb	Suicide plasmid	Keep in our lab
pACYCDuet-1	Co-expression system plasmid	Keep in our lab
pUC19-P3b-m	Synthesize the P3-B binding site mutant plasmid	IGEbio
pNV18-P3egfp	pNV18 *with promoter P3 and egfp, reverse* Kan^r	This study
pNV18-Egfp	pNV18 *with egfp,* Kan^r	This study
pNV18-Egfp- reverse	pNV18 *with egfp, reverse* Kan^r	This study

pNV18-P3egfp-R-1	pNV18 *with 172 bp P3 promoter deleted 10 bp from the 5′ terminus and egfp reverse* Kan^r	This study
pNV18-P3egfp-R-2	pNV18 *with 162 bp P3 promoter deleted 20 bp from the 5′ terminus and egfp reverse* Kan^r	This study
pNV18-P3egfp-R-3	pNV18 *with 152 bp P3 promoter deleted 30 bp from the 5′ terminus and egfp reverse* Kan^r	This study
pNV18-P3egfp-R-4	pNV18 *with 142 bp P3 promoter deleted 40 bp from the 5′ terminus and egfp reverse* Kan^r	This study
pNV18-P3egfp-R-5	pNV18 *with 132 bp P3 promoter deleted 50 bp from the 5′ terminus and egfp reverse* Kan^r	This study
pNV18-P3egfp-L-1	pNV18 *with 152 bp P3 promoter deleted 30 bp from the 3′ terminus and egfp reverse* Kan^r	This study
pNV18-P3egfp-L-2	pNV18 *with 122 bp P3 promoter deleted 60 bp from the 3′ terminus and egfp reverse* Kan^r	This study
pNV18-P3egfp-L-3	pNV18 *with 92 bp P3 promoter deleted 90 bp from the 3′ terminus and egfp reverse* Kan^r	This study
pNV18-P3egfp-L-4	pNV18 *with 62 bp P3 promoter deleted 120 bp from the 3′ terminus and egfp reverse* Kan^r	This study
pNV18-P3egfp-L-5	pNV18 *with 32 bp P3 promoter deleted 150 bp from the 3′ terminus and egfp reverse* Kan^r	This study
pET32a-Narl	pET-32a with gene coding for NarL from the strain P14 for	This study

	expression and purification of NarL protein Amp^r	
pACYCDuet-1-Narl-P3Egfp	pACYCDuet-1 with *promoter P3 and egfp reverse and narl*	This study
pACYCDuet-1-P3Egfp.	pACYCDuet-1 with *promoter P3 and egfp reverse*	This study
pNV18-cassette	Antibiotic cassete for suicide plasmid kan^r and CM^r	This study
pK18mobsacb-Narl	Knock out the gene *narl* in *Rhodococcus* sp. P14, CMr	This study

Table S2. List of primers used in this study.

Name	Sequences
P302 L up	CCCAAGCTTCGGACCGGCACGGGGCAGCGGGGGT
P304 L up	CCCAAGCTTTCGTTCGCTGGTGTGATGCCCCTCA
P305 L up	CCCAAGCTTACGGCTGACAGGCTGAACTCACTCC
P300JD R up	CCCAAGCTTTCGGCGTACGCATCACCACATCGAC
P301 R do	GCCCTTGCTCACCATGTGAGTTCAGCCTGTCAGCCGTTAG
P301-EGFP up	TGACAGGCTGAACTCACATGGTGAGCAAGGGCGAGGAG
P302 R do	GCCCTTGCTCACCATCCTGTCAGCCGTTAGAGTGAGGGGC
P302-EGFP R up	ACTCTAACGGCTGACAGGATGGTGAGCAAGGGCGAGGAG
P303 R do	GCCCTTGCTCACCATGTTAGAGTGAGGGGCATCACACCAG
P303-EGFP R up	ATGCCCCTCACTCTAACATGGTGAGCAAGGGCGAGGAG
P304 R do	GCCCTTGCTCACCATGGGGCATCACACCAGCGAACGAAAG
P304-EGFP R do	CGCTGGTGTGATGCCCCATGGTGAGCAAGGGCGAGGAG
P305 R do	GCCCTTGCTCACCATACCAGCGAACGAAAGTTGTCCACCT
P305-EGFP R do	AACTTTCGTTCGCTGGTATGGTGAGCAAGGGCGAGGAG
P300JD-EGFP do	GCTCTAGATTACTTGTACAGCTCGTCCATGCCG

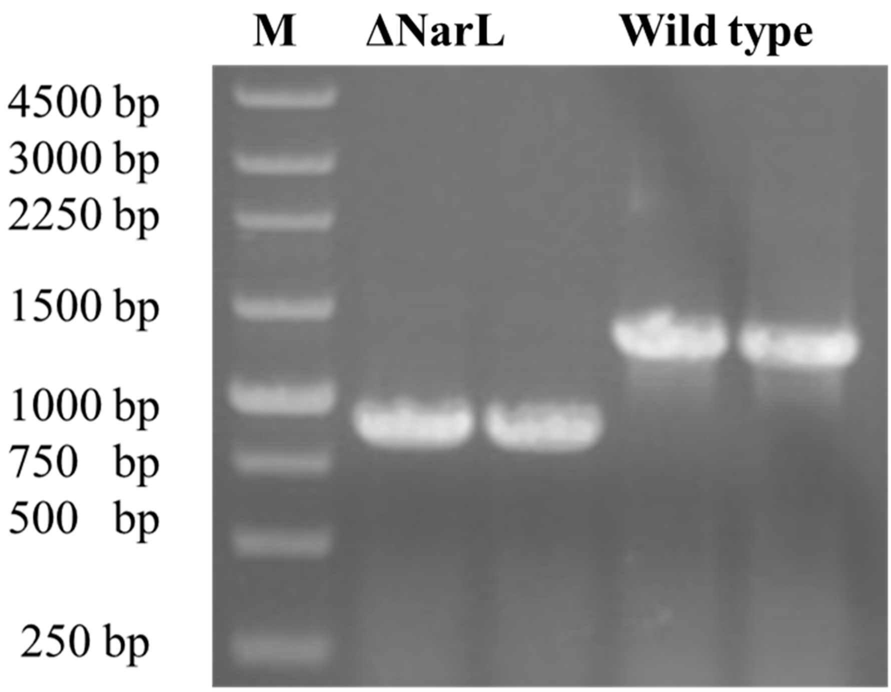

Figure S1. The PCR results of *narl* gene in wild type and ΔNarL. M was the marker 250 bp DNA ladder (TaKaRaA. The *narl* gene had deletion 555 bp in ΔNarL.

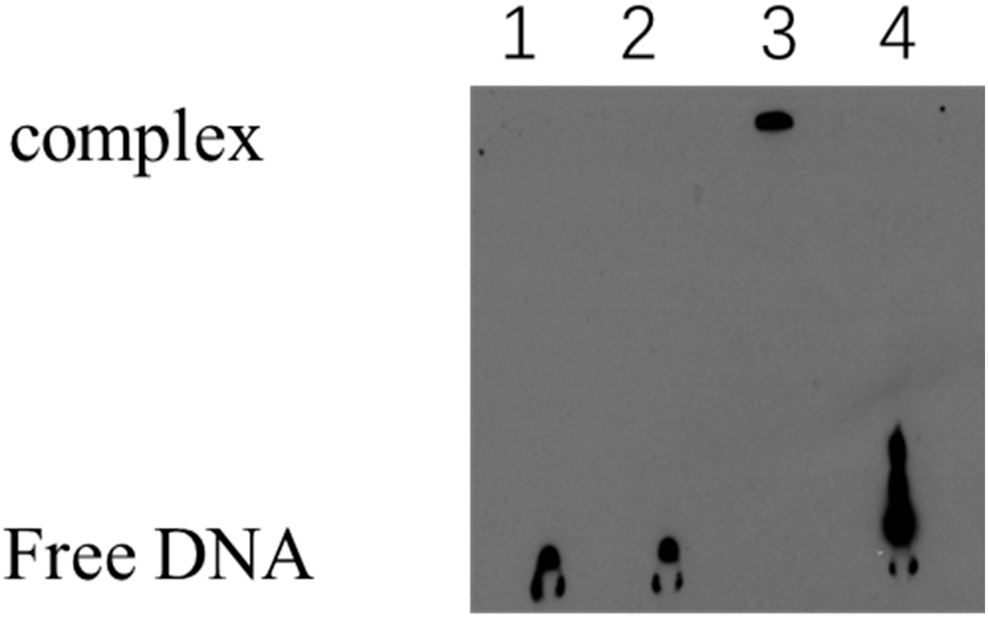

Figure S2. Competition experiments for the shift assay of P3-B and NarL. Lane 1 is P3-B (0.1 μM), lane 2 is P3-B (0.1 μM) and BSA (1 μM), lane 3 is P3-B (0.1 μM) and NarL (0.5 μM), and lane 4 is P3-B (0.1 μM), P3-B (unlabeled with biotin 15μM) and NarL (0.5 μM).

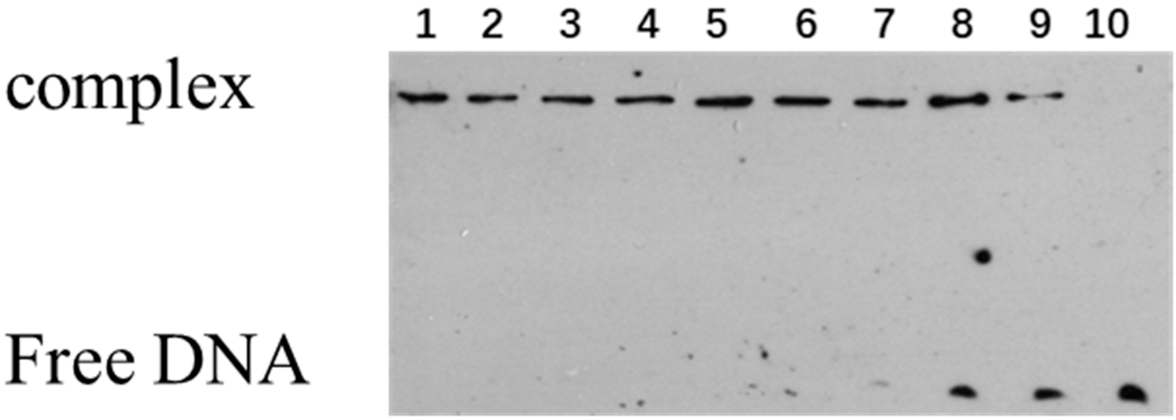

Figure S3. Effects of PAHs on NarL-DNA binding affinity. Lane 1 is DNA and NarL; lane 2 is DNA, NarL, and acetone; lane 3 is DNA, NarL, and biphenyl; and lane 4 is DNA, NarL, and hydroxy-biphenyl. Lanes 5, 6, and 7 are same as lane 2, 3, and 4, respectively. Lane 8 is DNA (excessive) and NarL; lane 9 is DNA (excessive), NarL, and hydroxy-biphenyl; and lane 10 is DNA. Comparing lanes 2–4 with lanes 5–7, samples were added in a different order. In lane 2–4, the DNA and protein were incubated first, then the PAHs were added. In lanes 5–7, PAHs and protein were incubated first, then DNA was. Neither of them werwe able to break the complex.

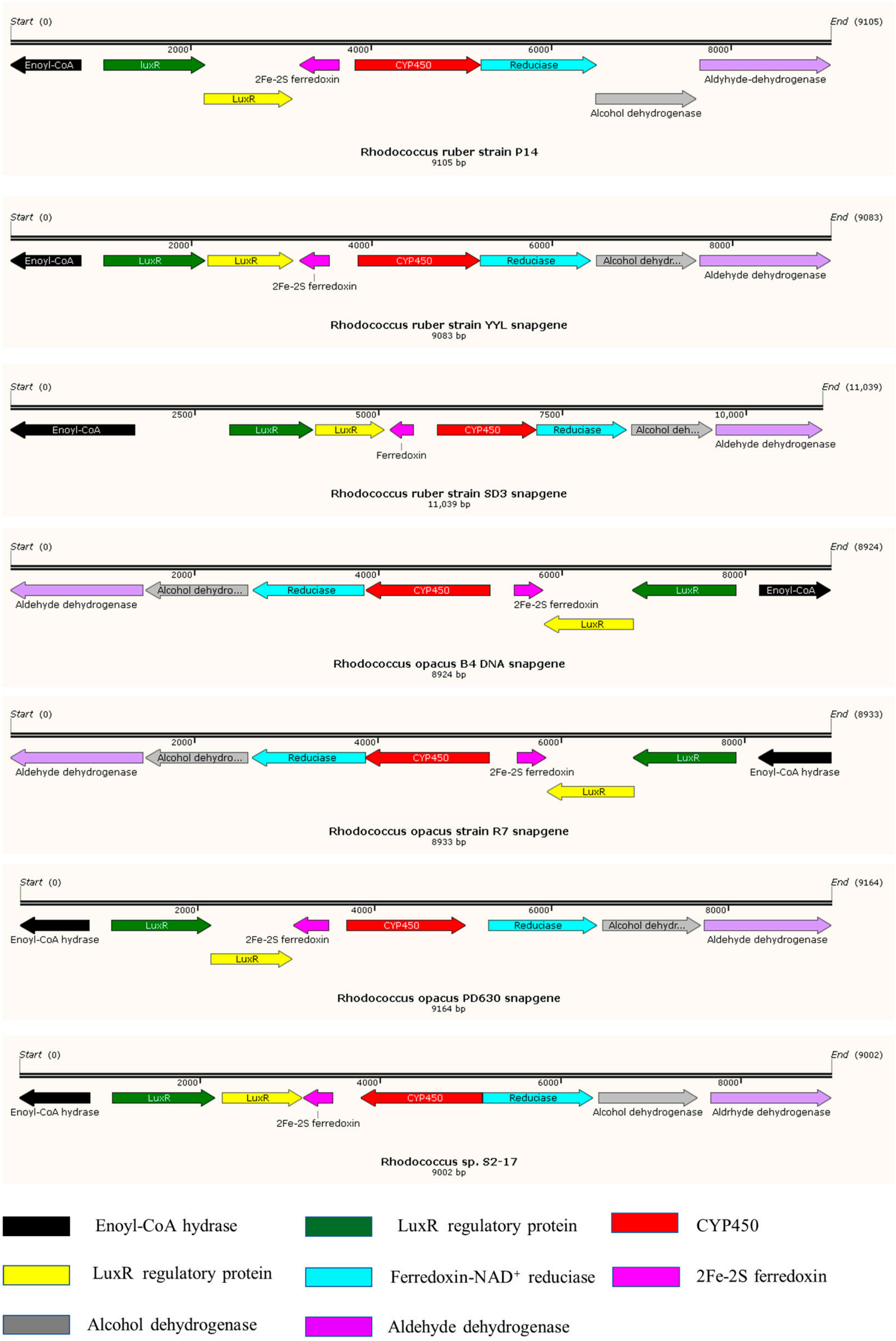

Figure S4. The conserved gene cluster consisting of CYP450 in the *Rhodococcus* genus.

A

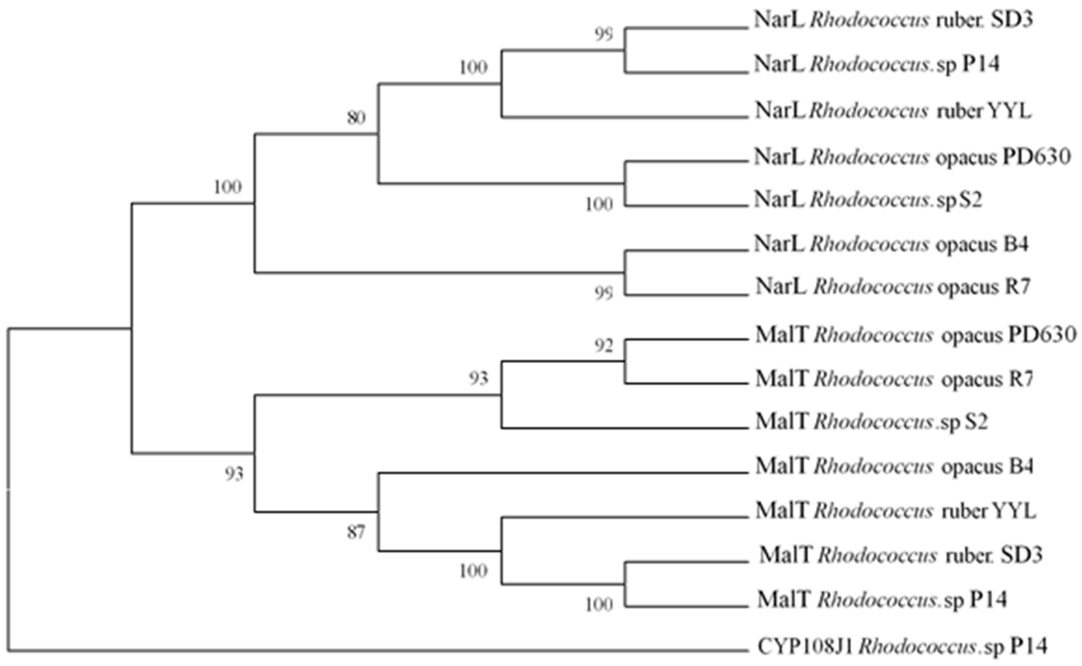

B

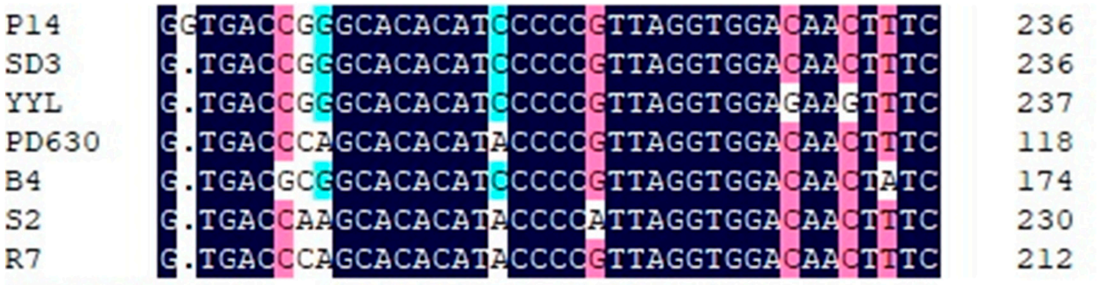

Figure S5. (**A**) Phylogenetic tree of NarL and MalT of *Rhodococcus* sp. P14 obtained from alignment with related proteins from other microorganisms. The numbers on branches refer to the percentage confidence, estimated by a bootstrap analysis with 1000 replications. (**B**) Alignment of the binding sequences of NarL with other similar sequences.
